# Are individual differences in auditory processing related to auditory distraction by irrelevant sound? A replication study

**DOI:** 10.3758/s13421-019-00968-8

**Published:** 2019-07-30

**Authors:** Emily M. Elliott, John E. Marsh, Jenna Zeringue, Corey I. McGill

**Affiliations:** 1 Department of Psychology, Louisiana State University, Baton Rouge, LA 70803 USA; 2 University of Gävle, Gävle, Sweden; 3 University of Central Lancashire, Preston, UK

**Keywords:** Replication, Auditory distraction, Individual differences, Serial recall

## Abstract

Irrelevant sounds can be very distracting, especially when trying to recall information according to its serial order. The irrelevant sound effect (ISE) has been studied in the literature for more than 40 years, yet many questions remain. One goal that has received little attention involves the discernment of a predictive factor, or individual difference characteristic, that would help to determine the size of the ISE. The current experiments were designed to replicate and extend prior work by Macken, Phelps, and Jones (*Psychonomic Bulletin & Review, 16*, 139–144, 2009), who demonstrated a significant predictive relationship between the size of the ISE and a type of auditory processing called global pattern matching. The authors also found a relationship between auditory processing involving deliberate recoding of sounds and serial order recall performance in silence. Across two experiments, this dissociation was not replicated. Additionally, the two types of auditory processing were not significantly correlated with each other. The lack of a clear pattern of findings replicating the Macken et al. (*Psychonomic Bulletin & Review, 16*, 139–144, 2009) study raises several questions regarding the need for future research on the characteristics of these auditory processing tasks, and the stability of the measurement of the ISE itself.

Every day, people are faced with tasks they must complete, coupled with distractions that may hinder the completion of those tasks. Studies show that, generally, auditory distractions, or irrelevant sounds, are detrimental to serial recall performance (e.g., Colle & Welsh, 1976; Jones & Macken, 1993; Salamé & Baddeley, 1982). Individuals cannot avoid the detrimental effects of these sounds, even when they are told that the sounds are not relevant to any portion of the task. In the laboratory, the size of the irrelevant sound effect (ISE) is often determined by the comparison of serial recall performance in silent trials and irrelevant sound trials (e.g., Ellermeier & Zimmer, 1997; Elliott, 2002; Macken, Phelps, & Jones, 2009). Convincing evidence suggests that the ISE is associated with preattentive sequential streaming—with the degree of disruption of serial recall increasing with the acoustic difference between successive stimuli within the sound until the point of perceptual fission. When the difference between successive stimuli becomes too large, the magnitude of disruption diminishes because the perceptual system partitions them into separate streams (Jones, Alford, Bridges, Tremblay, & Macken, 1999; Jones & Macken, 1995a, 1995b; Macken, Tremblay, Houghton, Nicholls, & Jones, 2003). According to a prominent account, the preattentive processing of order in the sound conflicts with the deliberate use of order cues involved in serially rehearsing to-be-remembered material (Jones & Tremblay, 2000). However, despite advances in understanding the mechanisms underpinning disruption from more than 40 years of research in this area, it has been difficult to determine a measure that predicts the degree of impact that sequences of irrelevant sound have on an individual’s performance. The present experiments explore this issue.

## Individual differences in auditory processing

Macken et al. (2009) conducted an important ISE and individual differences study, and concluded that one key to predicting the size of the ISE was the way that individuals process sounds. The Macken et al. study investigated two methods of auditory processing: deliberate sequence recoding and global pattern matching. The use of these two methods of auditory processing was influenced by the related work of Foxton et al. (2003).^1^

**Fig. 1.**
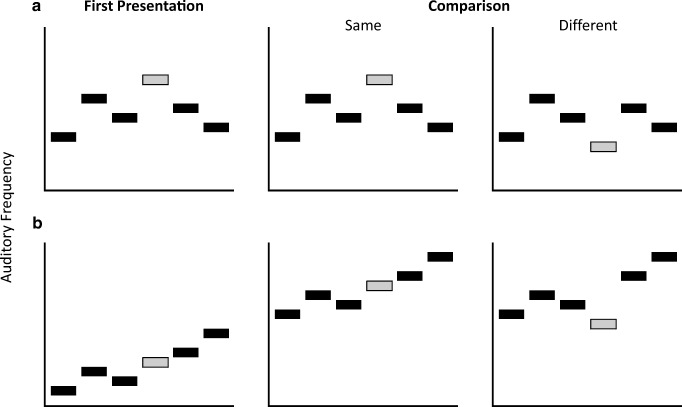
Based upon the figure used in Macken et al., 2009. Same and different stimuli in comparison to the first presentation in the global pattern matching task (**a**), and in the deliberate recoding task (**b**)

In the global pattern-matching task of the Macken et al. (2009) study, participants listened to two sets of tones and were asked to determine if they were the same or different (see Fig. 1 for an illustration of the different auditory conditions). The first set was then compared with the second set. If the comparison set was “different,” only one tone was different in relation to the first set. If it was a “same” trial, then all of the notes were exactly the same between the first and the comparison sets. The deliberate sequence recoding task was very similar, except that the sets of tones in both “same” and “different” trials in the comparison set were shifted either higher or lower in frequency. Furthermore, on “different” trials, one of the notes was displaced upwards or downwards compared with its relative position in the first set. This type of processing required the participant to engage in deliberate recoding, to be able to determine if the overall melody was the same or different, given that the entire comparison sequence was shifted in frequency.

Fifty undergraduate students participated in part one of the Macken et al. (2009) study, and 37 were retained in the final analyses. Participants were asked to complete 32 trials in both the deliberate recoding and global pattern-matching tasks. Half of the trials in each task included concurrent articulation. They found that concurrent articulation decreased performance on the deliberate recoding task to chance levels (*M* = 50.34%), but not the global pattern-matching task (60.98%).

Macken et al. concluded that this pattern of results supported the view that global pattern matching could be undertaken without direct recourse to intentional order-based processing, since it was impaired less by concurrent articulation than the sequences that required deliberate recoding. However, it should be noted that 13 of the original participants were not included in these analyses. The authors stated, “Participants performing close to chance on one task and close to ceiling on the other were excluded in order to avoid the possibility that any dissociation in performance across the two tasks could be attributed merely to floor or ceiling effects in one or the other” (Macken et al., 2009, p. 141).

Thirty-two of the participants from part one came back at a later date and completed a typical serial recall task within the ISE paradigm, using the same tones from part one as the irrelevant sound. The ISE was measured with a difference score by subtracting performance on the serial recall task in silence minus performance in the presence of the tones, referred to as “disruptibility.” The disruptibility measure and the measure of serial recall performance in quiet were entered into two separate regression analyses with the two sequencing conditions as predictors. A significant beta weight was only reported in the global pattern-matching condition for disruptibility, and not the deliberate recoding condition. However, the deliberate recoding condition did significantly predict serial recall performance in quiet, while the global pattern-matching condition did not.

To date, the Macken et al. (2009) study is the only known published study to suggest that an individual differences factor—namely, the passive processing of sounds—can predict the size of the ISE. On the face of it, this finding harmonizes with the view that the capability of automatically processing the order of sounds is intrinsically related to the degree of disruption such sounds produce to serial recall through the perceptual streaming processing (e.g., Macken et al., 2003). Specifically, the passive automatic processing of sound order, reported by Macken et al. (2009) could be a proxy for the processing of mismatch between representations of successive tokens that yields information about the order of elements, that in turn disrupts the deliberate serial recall process (Jones & Tremblay, 2000).

However, given the critical importance of replication in the field of psychology (e.g., Open Science Collaboration, 2015; Shrout & Rodgers, 2018), expanding a prospective sample beyond the original 32 participants and including additional conditions and measures would provide a larger and more diverse investigation of individual differences in the effects of auditory distraction on serial recall. Thus, the current study built upon this seminal study of individual differences in the ISE, and two experiments were conducted. Importantly, the same sound stimuli for the auditory sequencing tasks from Macken et al. (2009) were used in both of the experiments. Experiment 1 represented a conceptual replication, and Experiment 2 was designed to be as direct of a replication as possible (see Shrout & Rodgers, 2018, p. 492, for a discussion of types of replication studies).

## Experiment 1

In Experiment 1, we extended the original study by Macken et al. (2009). We increased the overall sample size, and added several measures. Digit span was included for an independent measure of serial recall performance, and we added measures of complex span to assess working memory capacity. Furthermore, Raven’s Advanced Progressive Matrices (RAPM) was included to assess general fluid intelligence, and finally, a visual measure of attention control, the antisaccade task, was administered.

While some research suggests that working memory capacity (WMC) measures correlate with forms of auditory distraction that are different from the classical ISE, such as attentional capture (produced by an unexpected change within a sequence of otherwise predictable events, i.e., the auditory deviant effect; e.g., Hughes, Hurlstone, Marsh, Vachon, & Jones, 2013; Marsh, Vachon, & Sörqvist, 2017; Sörqvist, 2010), other researchers have found conflicting results (Körner, Röer, Buchner, & Bell, 2017; Leiva, Andrés, Servera, Verbruggen, & Parmentier, 2016). However, the consensus is that WMC does not predict the size of the classical ISE (Beaman, 2004; Elliott & Briganti, 2012; Sörqvist, Marsh, & Nöstl, 2013). One critical issue with many of the prior studies investigating auditory distraction effects and individual differences in WMC has been in the measurement of WMC itself, although recent work by Körner et al. (2017) is an exception to this trend. Research indicates that the most valid, domain-general assessment of WMC is achieved through the use of multiple measures of WMC, as opposed to the use of a single measure, such as the operation span task alone (see Foster et al., 2015). Additionally, prior research has not included a separate assessment of attention control, without relying on a measure within the auditory distraction paradigm itself, such as the use of the auditory deviant effect as an index of attentional control processes. The current research sought to expand on the prior designs by including a full battery of WMC tasks, as well as an independent measure of attentional control.

In one visual attention capture task, the antisaccade task, participants are asked to not look at a flashing item on one side of the screen in order to see a target item on the opposite side of the screen. This tests the individual’s ability to avoid attention capture (Kane, Bleckley, Conway, & Engle, 2001). Performance on the antisaccade task is positively related to another measure of attention control, WMC (Chein & Weisberg, 2014).

In fact, WMC has been shown to predict an individual’s performance in various cognitive tasks, including showing consistent and positive relationships with general fluid intelligence, as measured by Raven’s Advanced Progressive Matrices (RAPM; Kane et al., 2004). There are several different complex span tasks used to measure WMC. The general model for each of these tasks is to remember a list of items while completing a distractor task in between each item and then later serially recalling the list of to-be-remembered items. The distractor task could be, for example, working a math problem, determining symmetry of an image, determining if an image is mirrored, or determining if a sentence makes sense. The to-be-remembered items can be, for example, a list of numbers or letters, the placement of blocks on a grid, or the direction of arrows. These tasks measure an individual’s ability to remember those target items while completing unrelated tasks in between. According to the findings of Foster et al. (2015), complex span tasks can be shortened without substantially reducing their predictive validity. The present study used one block of each of the three complex span tasks chosen in the Foster et al. study, to test the claim regarding the validity of the shortened tasks.

Based upon prior research with the WMC tasks, the antisaccade task, and the ISE, we hypothesized that since WMC does not always reliably predict the size of the ISE, neither would the antisaccade task. However, it was expected that the WMC scores would correlate with the antisaccade scores. Thus, measures that were predicted to correlate with the ISE were used, such as performance in the global pattern-matching auditory sequencing task, as well as measures that were not predicted to correlate, such as WMC. This method was useful in confirming which measures correlate with each other, even if they did not correlate with the size of the ISE. Additionally, we expected to replicate the correlations found in prior work by Foxton et al. (2003), demonstrating a relationship between performance on both types of the auditory sequences and RAPM.

To determine an appropriate sample size, we used G*Power 3 (Faul, Erdfelder, Buchner, & Lang, 2009), and we entered in the raw correlations from the original study by Macken et al. to find the post hoc power level of the regression analyses that were run in the original study. As expected, both of them were powered sufficiently (effect size *f*^*2*^ = .52, power = .94 for the prediction of the ISE by global pattern-matching performance and effect size *f*^*2*^ = .52, power = .95 for deliberate recoding performance). However, we were also interested in expanding beyond the original analyses, and the current design included several individual difference measures. Thus, we planned to recruit 100 participants.

### Method

#### Participants

One hundred and two undergraduate students from Louisiana State University participated in the study for psychology course credit or extra credit. The 20 males and 81 females (*M*_age_ = 20.21 years, range: 18–36 years) were native English speakers with normal hearing and corrected-to-normal vision. One male participant chose not to report his age. The participants completed five tasks in one session, lasting between 90 and 120 minutes. Participants were asked to read and sign a consent form before the start of the experiment and were assured they could leave the experiment at any time. Two participants did not meet the exclusion criteria listed above, and were not included in data analyses.

#### Materials

##### Working memory capacity tasks

The tasks used to measure WMC were taken from Foster et al. (2015): operation span, symmetry span, and rotation span. Each of the tasks consisted of a sequence of to-be-remembered items with intermittent distractors, followed by a recall prompt. The sequences consisted of anywhere from two to seven to-be-remembered items, depending upon the task. One block of each of the three tasks was administered, with one trial of each list length randomly presented in each task.

The first task was operation span (OSPAN), which consisted of letters as the to-be-remembered items and math problems as the distractor task. The participants solved a math problem and then were shown a letter. This sequence was repeated from three to seven times in a random order. After the sequence, the participants were asked to serially recall the letters they were shown during the sequence.

The second task was the symmetry span (SymSPAN) task. In this task, participants were shown a shape and were asked to decide whether it was symmetrical about a vertical axis. Once they answered, they were shown a 4 × 4 grid, with one of the squares highlighted in red. The participants were asked to remember the location of each highlighted square. After the sequence was repeated two to five times in a random order, the participants were shown a blank grid and were asked to recall the highlighted squares they saw throughout the duration of the trial, in order.

The final WMC task was the rotation span (RotSPAN) task. In this task, participants were shown a letter of the alphabet and were asked to determine whether the letter was presented correctly or if it was a mirrored image. Next, participants were shown either a short or long arrow pointed in one of eight possible directions. The letter–arrow sequence was repeated two to five times in a random order. At the end of each trial, participants were asked to recall the direction and size of each arrow presented in order.

##### Raven’s advanced progressive matrices

Participants were presented with a matrix of geometric patterns, with one part missing (Raven, Raven, & Court, 1998). Up to eight response options were given at the bottom of the screen. Using the mouse, participants selected the option that would complete the pattern. There were three blocks of 12 problems, totaling 36 problems, and the items increased in difficulty across each block. A maximum of 5 min was allotted for each block, totaling 15 min.

##### Attention control task

An antisaccade task modeled from Kane et al. (2001) was used to measure attention control. Participants began the trial by pressing any key. Immediately, a fixation cross appeared in the center of the screen for anywhere from 200 ms to 2,200 ms. The screen was then blank for 50 ms before an equals sign (“=”) appeared on either the left or right side of the screen and flashed twice for 100 ms with a 50-ms blank screen in between the flashes. After another 50-ms blank screen, a “P,” “B,” or “R” flashed on the opposite side of the screen for 100 ms. Immediately after the letter was shown, an “H” was shown for 100 ms, followed by an “8” until the participant provided a response. The participants were asked to report which letter they saw, using a key-press response. Participants completed the task at their own pace. There were six practice trials, followed by 24 critical trials.

##### Auditory sequencing tasks

The auditory sequence-processing task was recreated from Macken et al. (2009) using the sound files from the original experiment. There were two types of sequence processing involved in the task: global pattern matching and deliberate recoding. As a reminder, in the deliberate recoding condition, if the comparison sequence was different from the standard, one of the tones would be either higher or lower by a two-step change; additionally, the overall frequency of the tones was changed. The result of the two-step change to the comparison sequence altered the general “melody” of the sequence. Because the entire comparison sequence for both “same” and “different” trials was additionally changed in frequency in the deliberate recoding condition, a participant could not rely upon the actual pitches to make the same/different judgment. The participant had to be able to identify the change in the melody without reference to the original pitches to answer correctly.

Using the auditory stimuli from Macken et al., the tones presented in the tasks spanned the octave and were divided into seven equally spaced logarithmic steps, starting at 250 Hz. Each tone was 250 ms in length, with 20 ms rise and fall times. Each tone sequence was 1,500 ms, separated with 1,000 ms of silence.

There were two practice trials for both auditory sequence tasks, and there were two practice trials for the trials with concurrent articulation, for a total of four practice trials. Immediately following the practice were 32 test trials in each of the types of processing sequences, for an overall total of 64 test trials. Participants were asked to complete half of the trials in silence (e.g., 16 in each type). In the other half of the trials, participants were asked to say the word *the* out loud at a rate of twice per second. Prior to each trial, the participants were told whether the following trial would be a “same octave” or global pattern matching, or a “different octave” or deliberate recoding, as well as whether or not they were to use concurrent articulation. Participants worked at their own pace, and indicated their answers on a computer keyboard.

##### Digit span task

The digit span task, used to measure serial recall abilities, was taken from Elliott (2002). In this task, participants were shown lists of digits on a computer screen at a rate of one digit per second. The digit lists included the set of numbers 1 through 9, and digits were not used more than once in the same list. Each participant started with a block of four lists, each of which were three digits in length. If the participant correctly recalled two or more of the lists, the list length increased by one, with an additional four lists. If participants failed to correctly recall two lists, or if the participant completed the trials of list-length nine, the program ended. The highest list length with at least two of the four lists correctly recalled was recorded as the participant’s digit span.

##### Irrelevant sound task

This task was also adopted from Macken et al. (2009). Participants were shown lists of eight digits in a random order from the set of digits 1 to 9, with no repeated digits, at a rate of one per second. Then a blank screen appeared for 10 seconds before participants were cued to recall. There were a total of four practice trials: two performed in silence and two performed in the presence of irrelevant sound. There were 36 test trials in this task, 18 in silence and 18 trials with the tone sequences from the auditory sequencing task played continuously. Participants were told to ignore the tones and were assured they would not be questioned about them.

#### Procedure

Participants were run individually and the tasks were presented in a fixed order, all of which were completed in one session. The original task order included: a demographics questionnaire, RAPM, Ospan, SymSpan, RotSpan, antisaccade task, digit span, auditory sequencing task, and then the ISE task.

The instructions for the sequencing task were made as simple as possible, to help explain the patterns of the notes to those without music experience:

*“In this experiment, you will be asked to listen to several pairs of tone sequences and make same/different judgments on them. . . . You will be told before each trial whether that particular trial will involve a SAME OCTAVE or DIFFERENT OCTAVE comparison. After each pair of tone sequences play, you will be asked to press ‘S’ on the keyboard if you thought the sequences were the same, and ‘D’ if you thought the sequences were different.”*

The “same octave” instructions corresponded with the global pattern-matching condition, although the participants were not told that terminology, whereas the “different octave” instructions were considered deliberate recoding trials.

After 23 participants had completed the tasks, we observed that participants found the tasks very difficult, based on spontaneous comments made in the experimental session, and that performance on the auditory sequencing task was lower than expected. In an effort to motivate performance and minimize potential effects of fatigue, the sequencing task was moved to the first position in the task order, with all other tasks remaining in the same position.

#### Scoring

In the WMC tasks, participants received one point for each item they correctly recalled, regardless of whether the entire list was correct. Because the tasks had different list lengths, and thus different possible total scores, the scores for each task were individually converted to *z* scores. The *z* scores for each task were then averaged together to create a composite WMC score.

In the antisaccade task, the percentage of correctly identified letters was computed for each participant, while the final score from the RAPM task was the total number of correct responses across the three blocks. For the digit span task, participants received an integer span score, which was the highest list length in which they correctly recalled 50% of the trials. There were four separate scores for each individual for the auditory sequencing task. Participants were scored on the percentage of trials correctly completed in each of the four conditions: deliberate recoding with and without concurrent articulation, and global pattern matching with and without concurrent articulation. Finally, the size of the ISE was calculated by subtracting the proportion of correct trials during simultaneous irrelevant sound from the proportion of correct trials in silence.

### Results

Following the analysis protocol of Macken et al. (2009), and to avoid concerns related to floor effects in the conditions of the sequencing task, the participants that scored at or below chance on both of the “without concurrent articulation” conditions were excluded (*n* = 7). Also, as no participants scored 90% or higher in the global pattern-matching condition, we were not concerned about ceiling effects, as discussed in Macken et al.

Descriptive statistics and correlational analyses for the three WMC measures are presented in the Appendix, and Tables 1 and 2 present the descriptive statistics and correlations for all measures, including the WMC composite score. As expected, the composite WMC measure and performance on the antisaccade task were significantly correlated. Also, as noted in prior work by Elliott and Cowan (2005), there was a significant correlation between the ISE difference score and digit span. However, the only significant correlation to emerge from the sequencing task was the global pattern-matching condition without concurrent articulation and digit span. There were no significant correlations between the auditory sequencing tasks and the ISE. Finally, the correlation between digit span and the WMC composite score did not reach statistical significance, but was in the expected direction (*r =* .17).

The same repeated-measures 2 × 2 ANOVA as in Macken et al. was conducted to determine if the conditions in the sequencing task were significantly different. There were main effects of both sequencing condition, *F*(1, 92) = 22.10, *MS*_*e*_ = .01, *p* = .001, η_p_^2^ = .19, and concurrent articulation, *F*(1, 92) = 39.68, *MS*_*e*_ = .01, *p* = .001, η_p_^2^ = .30. Performance was higher in global pattern matching (*M* = .58, *SE* = .01) than in deliberate recoding (*M* = .52, *SE* = .01), and performance was higher without concurrent articulation (*M =* .59, *SE* = .01) than with concurrent articulation (*M* = .51, *SE* = .01). There was also a significant interaction between the two factors, *F*(1, 92) = 15.09, *MS*_*e*_ = .01, *p* = .001, η_p_^2^ = .14. Performance was highest overall in the global pattern-matching task without AS (*M* = .63, *SE* = .01).

As in Macken et al. (2009), a one-sample *t* test was conducted to determine if the auditory sequencing conditions were different from chance. Both conditions without concurrent articulation, deliberate recoding, *t*(92) = 3.77, *p* = .001, and global pattern matching, *t*(92) = 12.05, *p* = .001, were significantly different from chance. Also, global pattern matching with concurrent articulation, *t*(92) = 2.18, *p* = .031, was significantly different from chance, while deliberate recoding with concurrent articulation was not significantly different from chance, *t*(92) = .614, *p* = .541. These results replicated Macken et al. (2009) as well.

Finally, the results of two regression analyses were nonsignificant. Neither performance in the global pattern-matching task without concurrent articulation nor the deliberate recoding task without concurrent articulation predicted the size of the ISE or performance on the digit span task, which does not replicate the results of Macken et al. (2009). The analysis predicting the size of the ISE was not significant, *F*(2, 90) = 0.067, *p* = .935, standardized beta β = .02, *p* = .834, for the global-pattern matching condition, and β = .0, *p* = .776, for the deliberate recoding condition. The analysis predicting digit span performance was also not significant overall, *F*(2, 90) = 2.21, *p* = .115. However, the standardized beta, β = .216, *p* = .039, for the global pattern-matching condition was significant, but it was not significant for the deliberate recoding condition, β = −.03, *p* = .77. These results did not replicate Macken et al.

### Discussion

The study of Macken et al. (2009) is the only one to date that has found evidence of a variable that predicts the size of the ISE. However, the current experiment was not able to replicate those findings. The study reported here used the same sound files for the auditory sequencing task as the original Macken et al. study. Additionally, the current Experiment 1 had a significantly larger sample size than the original study. However, the prior studies using auditory sequencing tasks by Foxton et al. (2003) and Macken et al. both indicated significant positive correlations between performance on the two types of auditory sequences, without concurrent articulation (*r* = .45 and .62, respectively), and this significant correlation did not occur in Experiment 1 (*r* = .06).

Importantly, this study did replicate Macken et al. (2009), along with other studies, in regard to the size of the ISE. Prior studies have shown about a 10% decrease in the absolute measure of performance comparing the presence of irrelevant sound versus silence (e.g., Elliott & Briganti, 2012; Macken et al., 2009); additionally, Ellermeier and Zimmer (2014) demonstrated in their review that when tones were used as the irrelevant sound, as opposed to speech, the size of the disruption was less (see Table 1). Our findings are consistent with an ISE that was caused by tonal stimuli. The current study also succeeded in replicating Elliott and Briganti (2012) and others (e.g., Beaman, 2004; Sörqvist, 2010) in that WMC was not found to significantly correlate with the size of the ISE (see also Sörqvist et al., 2013). Furthermore, a replication of Foster et al. (2015) was found with significant and positive correlations among the three measures of WMC, and also with the Raven’s task. The current Experiment 1 presented one block of each of the complex span tasks as opposed to the traditional three-block tasks, following the recommendation of Foster et al.

**Table 1:** Descriptive Statistics in Experiment 1, *N* = 93

Task		Mean	*SD*	Min.	Max
Auditory sequencing	Deliberate recode with concurrent articulation	.51	.12	.13	.81
	Deliberate recode without concurrent articulation	.54	.11	.25	.88
	Global pattern match with concurrent articulation	.52	.09	.31	.81
	Global pattern match without concurrent articulation	.63	.11	.38	.88
Irrelevant sound effect		.09	.10	−.15	.31
Antisaccade		.64	.21	.17	1.0
Working memory capacity *z*-score average		.04	.70	−1.88	1.41
Digit span		6.90	1.21	4.0	9.0
Raven’s Advanced Progressive Matrices		25.58	3.63	17.0	34.0

However, unlike the prior work of Foxton et al. (2003), performance on RAPM in the current study did not show significant and positive correlations with the two types of auditory sequencing performance. The lack of significant correlations of the two types of auditory sequencing performance with either each other, or the RAPM task, motivated the undertaking of Experiment 2. As far as we can ascertain from the Method sections of the two prior works, the main difference between the current Experiment 1 and the studies of Foxton et al., and Macken et al. is that in the latter two, the trials were blocked for task type (i.e., deliberate recoding and global pattern matching), while in the current Experiment 1, trials were completely randomized. Therefore, one could speculate that the changes in methodology in the current Experiment 1 could be a reason for not replicating the results of Macken et al. (2009), and this observation was a further motivation for Experiment 2.

**Table 2:** Correlational Analyses from Experiment 1, *N* = 93

Task	1	2	3	4	5	6	7	8
1 Deliberate recode with concurrent articulation	—							
2 Deliberate recode without articulation	.06	—						
3 Global pattern match with concurrent articulation	.14	.04	—					
4 Global pattern match without articulation	−.11	.06	.03	—				
5 Irrelevant sound effect	.05	.03	.11	.02	—			
6 Digit span	.05	−.02	.07	.21*	.02	—		
7 Antisaccade	.18	.12	−.01	.08	−.09	.20	—	
8 Raven’s Advanced Progressive Matrices	.03	.02	.01	−.05	−.16	.02	.23*	
9 WMC *z* score	.12	.02	.02	−.01	−.04	.17	.29**	.21*

*p < .05. *p < .01

**Table 3:** Descriptive Statistics, Experiment 2, *N* = 69

Task		Mean	*SD*	Min.	Max
Auditory sequencing	Deliberate recode with concurrent articulation	.53	.11	.31	.75
	Deliberate recode without concurrent articulation	.57	.12	.25	.88
	Global pattern match with concurrent articulation	.54	.11	.25	.81
	Global pattern match without concurrent articulation	.71	.12	.44	1.00
Irrelevant sound effect		.09	.10	−.13	.37
Serial recall in silence		.67	.19	.12	1.00
Serial recall with tones		.58	.18	.10	1.00

**Table 4:** Correlational Analyses, Experiment 2, *N* = 69

Task	1	2	3	4	5
1 Deliberate Recode With Concurrent Articulation	–				
2 Deliberate Recode Without Articulation	−.04	–			
3 Global Pattern Match With Concurrent Articulation	.02	.09	–		
4 Global Pattern Match Without	.15	.12	.16	–	
5 Irrelevant Sound Effect	−.16	.20	−.04	.42***	–
6 Serial Recall in Silence	−.02	.16	.03	.23	.38*

**p < .001. *p = .001

Thus, in Experiment 2, the sequencing task was run again, this time with the tasks blocked, as in the Macken et al. (2009) study. It is possible that the randomized presentation of the deliberate recoding and global pattern-matching conditions contaminated performance across trial types in Experiment 1. For example, in global pattern-matching trials, one should not need to form a strategy. However, during the deliberate recoding trials, one has to think strategically about each individual tone in each set. The strategy formed for deliberate recoding trials could be interfering with performance on the global pattern-matching trials in the randomized version. If the pattern of results is changed between the auditory sequencing tasks and the ISE using the blocked sequencing tasks, this would indicate that strategic contamination was likely occurring in the randomized version.

## Experiment 2

To address the methodological concerns regarding the order of the sequencing tasks, Experiment 2 was conducted with a new sample of participants. This experiment represents the clearest attempt at a direct replication of the original Macken et al. (2009) work, including the use of the same sound sequences across the two tasks and the blocked presentation of the conditions in the auditory sequencing task. Participants completed all of the global pattern-matching trials in one block, with concurrent articulation trials randomly occurring on half of the trials. Participants then completed the deliberate recoding trials, again with concurrent articulation on half of the trials. Finally, participants completed the ISE task. The order of tasks was fixed, to limit strategic influences on the outcome and to match the original procedure of Macken et al. as closely as possible. Participants always completed the global pattern-matching block of trials first, thus limiting the application of deliberate recoding-style processing during the global pattern-matching sequencing task. No other tasks were completed during Experiment 2, as the focus was on the relationship of the auditory sequencing task and the ISE task, as a direct replication of the original study. The planned sample size was smaller than in Experiment 1, due to the reduction in measures used in the current experiment, but still more than double the number of participants recruited in the original study by Macken et al.

### Method

#### Participants

Eighty undergraduate students (*M*_*age*_= 20.21 years, range: 18–36 years) from Louisiana State University participated in the study for psychology course credit or extra credit. The participants were native English speakers with normal hearing and corrected-to-normal vision. The participants completed two tasks in one session, lasting approximately 30 minutes. Participants were asked to read and sign a consent form prior to the start of the experiment and were assured they could leave the experiment at any time.

#### Materials

All materials were the same as in Experiment 1 for the two tasks in this experiment.

#### Procedure

Participants completed the same auditory sequencing task and irrelevant sound task as in Experiment 1. The only change to the auditory sequencing task was that the conditions were presented in separate blocks of trials. All participants received the tasks in a fixed order, as described above.

### Results

Following the procedure of Macken et al. (2009) regarding floor and ceiling effects, eight participants were removed for having at or below chance performance on both auditory sequencing tasks. Additionally, three participants were removed for having performance levels at ceiling (90% or higher) on the global pattern-matching task, while these same participants performed at or below chance on the deliberate recoding task. Descriptive statistics and correlations are reported in Tables 3 and 4.

Again, following the analysis approach of Macken et al. (2009) and Experiment 1, a repeated-measures 2 × 2 ANOVA was used to analyze the four conditions in the sequencing task. There were main effects of both sequencing condition, *F*(1, 68) = 35.47, *MS*_*e*_ = .01, *p* = .001, η_p_^2^ = .34, and concurrent articulation, *F*(1, 68) = 62.52, *MS*_*e*_ = .01, *p* = .001, η_p_^2^ = .48. There was also a significant interaction between the two factors, *F*(1, 68) = 22.18, *MS*_*e*_ = .01, *p* = .001, η_p_^2^ = .25. The pattern of means was similar to the outcome of Experiment 1 and Macken et al.

As in Experiment 1, one-sample *t* tests were conducted to determine if mean performance in each of the sequencing conditions was significantly different from chance. All were significantly different from chance, deliberate recoding without concurrent articulation, *t*(68) = 4.553, *p* = .001, global pattern matching without concurrent articulation, *t*(68) = 14.238, *p* = .001, global pattern matching with concurrent articulation condition, *t*(68) = 3.123, *p* = .003, and finally, the deliberate recoding with concurrent articulation condition, *t*(68) = 2.145, *p* = .036. The finding that even deliberate recoding with concurrent articulation was significantly greater than chance differed from both Experiment 1 and Macken et al.’s original findings.

Finally, two separate regression analyses were conducted with the control conditions (no concurrent articulation) of global pattern matching and deliberate recoding predicting the size of the ISE and serial recall in silence (see Table 5). The analysis predicting the size of the ISE was significant overall, *F*(2, 66) = 8.147, *p* = .001, with a significant standardized beta, β = .398, *p* = .001, for the global pattern-matching condition. The analysis predicting serial recall in silence was not significant overall (*p* = .084).

**Table 5:** Results of the regression analyses predicting the size of the irrelevant sound effect and serial recall in silence, *N* = 69

Dependent variable	Predictors	β
Irrelevant sound effect	Deliberate recoding	.16
	Global pattern matching	.40*
Serial recall in silence	Deliberate recoding	.13
	Global pattern matching	.22

*p = .001

### Discussion

The importance of the blocked presentation of the auditory sequencing tasks, thus limiting the opportunity for strategic contamination, was examined in Experiment 2. The regression analyses did replicate the relationship between the ISE and performance in the global pattern-matching task from the Macken et al. (2009) study. However, the regression analyses with serial recall in silence did not lead to significant findings, unlike the Macken et al. study, in which performance in deliberate recoding was related to serial recall in silence. We did find one significant correlation between the size of the ISE and performance on the global pattern-matching auditory sequencing task; however, the two auditory sequencing tasks did not correlate with each other, which was also a concern in Experiment 1. Thus, while the order of the sequencing tasks does seem to play a role in the relationships among the variables, using a blocked format of presentation did not lead to a clear replication of the prior work. These findings, and their implications, will be discussed in relation to the outcome of Experiment 1 below.

## General discussion

Two experiments were conducted to examine individual differences in the size of auditory distraction effects with the goal of replicating and extending the original work of Macken et al. (2009). To briefly summarize the main manipulations, Experiment 1 expanded the range of measures used to include a valid and reliable battery-based assessment of WMC, an independent measure of attention control through the visual antisaccade task, a measure of general fluid intelligence (RAPM), digit span, auditory sequencing tasks, and the ISE. Additionally, both Experiments 1 and 2 used the original sound stimuli of Macken et al. for the auditory sequencing tasks and the ISE. Finally, Experiment 2 presented a more direct replication of Macken et al., and focused in narrowly on the relationships between the auditory sequencing task and the ISE, with a blocked presentation of the sequencing tasks.

The findings of these two experiments did not replicate Macken et al. (2009) overall. It is important to note that Experiment 2 did replicate the prediction of the size of the ISE by performance in the global pattern-matching auditory sequence task, but several other predicted relationships were not observed. Additionally, neither of the overall regression analyses were significant in Experiment 1. Even in the face of potential strategic contamination that would have affected the global pattern-matching auditory sequence task, the lack of a relationship between serial recall performance and performance in the deliberate recoding task was surprising.

Overall, it is of concern that levels of performance in the two auditory sequence tasks tended to be low. Additionally, following the protocol used in the original Macken et al. (2009) work, participants had to be removed from both experiments to deal with potential floor effects (particularly in the deliberate recoding task) and with ceiling effects in the global pattern-matching task. These problems with levels of performance suggest that a more thorough task analysis of the two auditory sequencing conditions is warranted. Based upon the previous findings of Macken et al., it was predicted that this auditory sequencing task would lead to a passive form of listening, but those results were difficult to reproduce in the current experiments.

However, the current study did not replicate the patterns of correlations that were observed in the original study. The observed power of the current Experiment 2 (the one with the closer design to that of the original study) was calculated to be .94 (effect size *f*^*2*^ = .22) for global pattern matching and .35 (effect size *f*^*2*^ = .05) for deliberate recoding. The variability in the observed effect sizes in the current study, as compared with the post hoc calculation of the original effect sizes (see Experiment 1), is consistent with a recent discussion on “planning power for a replication study” (Shrout & Rodgers, 2018, p. 493) and highlights the need for more studies on individual differences in the ISE. Meta-analysis in other areas of research have indicated that there is variability among effect sizes, and that effect sizes should be considered as a distribution, as opposed to a single, fixed number. Additionally, publication bias should be taken into consideration as well. Work by Anderson, Kelley, and Maxwell (2017) has provided a tool to calculate sample size which helps the researcher to take both uncertainty around the estimate of effect size and publication bias into account.

Furthermore, another consideration is the use of a difference score when studying the ISE, and whether a difference score influences the reliability of the measure. As mentioned above, previous research by Ellermeier and Zimmer (1997) demonstrated that the effects of irrelevant sounds were reliable within participants, using a test–retest design. However, the test–retest portion of the study included only 25 participants. Additionally, internal consistency reliability was reported at α = .55, which is a moderate level. Finally, a concern raised during the review process was that these reliability data from Ellermeier and Zimmer were obtained by comparing performance on a serial recall task in three auditory conditions, silence, pink noise, and irrelevant speech in a foreign language, while the current experiments used tones as the irrelevant sounds. Future research should address the issues of the type of irrelevant sound used within the paradigm and the test–retest reliability of the effects in larger samples of participants under different sound conditions of the ISE.

On the face of it, the finding of a relationship between the magnitude of the ISE and automatic encoding would seem to gel with the central role that the auditory streaming process plays in the ISE (Jones et al., 1999; Macken et al., 2003). However, the failure to replicate the key findings of Macken et al. (2009) may have connotations for the view that irrelevant sounds are automatically processed and therefore impossible to ignore even when participants are instructed to do so. One possibility as to why the replication of the relationship between the ISE and automatic encoding failed in the current study may be because only a few listening strategies are associated with susceptibility to the ISE. This is discussed in Billing and Carlyon (2016) in relation to deviant detection, but is nonetheless valid here. The authors demonstrated that focused attention promoted auditory streaming using both objective and subjective measures. The use of both measures provided important additional details on auditory streaming and under what conditions stream segregation was automatic. In some conditions, participants were presented with noises and tones and were instructed to either attend to the tones for a deviant detection task while ignoring noise, or to ignore the tones. Participants were further given instructions regarding focusing attention on one aspect of the task, or switching attention (for example, from the noises to the tones). The authors demonstrated the importance of focused attention to streaming, and also that attentional switches can reset it. These results were obtained in a task devoid of serial order processing; however, the views regarding the automaticity of disruption by auditory stimuli may need to be expanded. It should be noted here that the relationship between attention and streaming of sound that is task-irrelevant is far from clear, with Macken et al. (2003) providing evidence that streaming of irrelevant sound may indeed be preattentive and therefore independent of attention. The design of Billig and Carlyon’s (2016) study, coupled with the recent work of Hanczakowski, Beaman, and Jones (2017, 2018), may provide a new direction for future research in this area by incorporating both objective and subjective assessments of performance.

Of additional note, there are individual differences in some aspects of lower level auditory processing which suggest that auditory streaming processes might not be completely invariant. For example, there are age differences in the perceptual organization of sounds (Alain, Dyson, & Snyder, 2006), which include sound localization (Abel, Krever, & Alberti, 1990), and difficulties in determining the sequential order of sounds (Trainor & Trehub, 1989). Therefore, perhaps the global pattern-matching task does not, in fact, tap into the primitive perceptual organization processes that underpin the ISE. Complicating this view, however, is that there is some evidence that primitive processes—for example, fusion threshold in the context of sequential sound segregation—that have indeed been associated with the ISE (Jones et al., 1999), are related to higher order auditory and cognitive functions such as sentence perception from simultaneous sentences (Mackersie, Prida, & Stiles, 2001).

### Conclusions

Overall, across two experiments, the relationships among global pattern matching and deliberate recoding in auditory sequence processing with measures of serial recall and the ISE did not lead to consistent correlations. The prior work of both Foxton et al. (2003) and Macken et al. (2009) used small sample sizes for correlational research, which may contribute to the lack of a complete replication in the current work; thus, the importance of replication studies is highlighted here. Future research must be conducted to attempt to address these questions about individual differences in auditory distraction effects and the processes used in auditory sequence tasks. At present, a measure that predicts the magnitude of disruption-irrelevant sound has on an individual’s performance remains elusive.

#### Author note

Experiment 1 was originally presented at the 2016 Annual Meeting of the Southeastern Psychological Association in New Orleans, Louisiana.

#### Open practices statement

The data and materials will be made available upon request. The experiments reported here were not preregistered.

## Appendix

Descriptive statistics and correlational analyses were conducted first for the raw WMC complex span task measures (see Tables 6 and 7). There were significant correlations among the three complex span tasks. These correlational results justify the creation of the WMC composite score, because it demonstrates that the measures are significantly and positively related to one another. Additionally, the negative correlations between the processing measures and the recall measures in each task suggested that there were no systematic trade-offs between recall and processing (e.g., Unsworth, Redick, Heitz, Broadway, & Engle, 2009). Therefore, additional analyses with the processing scores were not included.

**Table 6:** Descriptive statistics for raw working memory capacity measures, *N* = 93

Task	Mean	*SD*	Minimum	Maximum
OSpanRecall	18.86	4.73	4	25
OspanErrors	1.71	1.52	0	7
SymSpanRecall	8.98	2.79	3	14
SymSpanErrors	1.01	1.42	0	9
RotSpanRecall	9.48	3.04	2	14
RotSpanErrors	1.51	2.17	0	14

**Table 7:** Correlational results from raw working memory capacity measures, *N=* 93

	1	2	3	4	5
1. OSpanRecall	—				
2. OSpanErrors	−.37**	—			
3. SymmSpanRecall	.29**	−.02	—		
4. SymmSpan Errors	−.08	.04	−.30**	—	
5. RotSpanRecall	.21*	−.21*	.31**	−.29**	
6. RotSpanErrors	−.06	.02	.08	.14	−.41**

***p* < .01. **p* < .05

## Footnotes

1 The terminology used by the two sets of authors differed, and the current work used the nomenclature of the Macken et al. (2009) work. Foxton et al.’s (2003) publication referred to the global pattern matching condition as a “local sequence,” because of the emphasis on “absolute pitch values,” while the deliberate sequence recoding condition was termed the “global sequence” to illustrate the importance of pitch patterns when making the same/different judgment.
